# The indispensable *whole* of work and population health: How the working life exposome can advance empirical research, policy, and action

**DOI:** 10.5271/sjweh.4130

**Published:** 2024-03-01

**Authors:** Yorghos Apostolopoulos, Sevil Sönmez, Matthew S Thiese, Lazaros K Gallos

**Affiliations:** 1 Prolepsis: Partners in Prevention Inc, Orlando, Florida, USA.; 2 University of Central Florida College of Business, Orlando, Florida, USA.; 3 Rocky Mountain Center for Occupational and Environmental Health, University of Utah School of Medicine and Weber State University, Salt Lake City, Utah, USA.; 4 DIMACS, Center for Discrete Mathematics & Theoretical Computer Science, Rutgers University, Piscataway, New Jersey, USA.

**Keywords:** exposome, complexity, occupational health, safety, and wellbeing, working life, whole of work and population health, working life exposome

## Abstract

**Objectives:**

The thesis of this paper is that health and safety challenges of working people can only be fully understood by examining them as *wholes* with interacting parts. This paper unravels this indispensable *whole* by introducing the working life exposome and elucidating how associated epistemologies and methodologies can enhance empirical research.

**Methods:**

Network and population health scientists have initiated an ongoing discourse on the state of empirical work-health-safety-well-being research.

**Results:**

Empirical research has not fully captured the totality and complexity of multiple and interacting work and nonwork factors defining the health of working people over their life course. We challenge the prevailing paradigm by proposing to expand it from narrow work-related exposures and associated monocausal frameworks to the holistic study of work and population health grounded in complexity and exposome sciences. Health challenges of working people are determined by, embedded in, and/or operate as complex systems comprised of multilayered and interdependent components. One can identify many potentially causal factors as sufficient and component causes where removal of one or more of these can impact disease progression. We, therefore, cannot effectively study them by an a priori determination of a set of components and/or properties to be examined separately and then recombine partial approaches, attempting to form a picture of the *whole*. Instead, we must examine these challenges as *wholes* from the start, with an emphasis on interactions among their multifactorial components and their emergent properties. Despite various challenges, working-life-exposome-grounded frameworks and associated innovations have the potential to accomplish that.

**Conclusions:**

This emerging paradigm shift can move empirical work-health-safety-well-being research to cutting-edge science and enable more impactful policies and actions.

Political, economic, and social institutions around the world have different views about work organization, especially the rights of and protections for working people. Over the years, these varied approaches have shaped the discourse around work and population health, safety and well-being, thereby, significantly influencing empirical research.

The well-being of working people is attributable to the confluence of diverse, multifactorial, and interacting, work and nonwork influences (1). However, empirical research has not fully captured the indispensable *whole* of work and population well-being – that is, the totality and complexity of relevant exposures and factors, their relationships, and emergent behaviors over the lifespan, not just during working years. Subsequently, this incomplete understanding of work-health-safety relationships continues, resulting in inadequate policies and actions. Therefore, it is time for a paradigm shift to create a greater understanding of the role of working life in population health and safety.

Herein, we: (a) explicate how traditional research impasses impede sufficient understanding of the *whole* of health and safety challenges of working people; (b) discuss how synergies of holistic epistemologies – grounded in complexity and exposome sciences – can improve current understanding of working life and well-being; (c) introduce a comprehensive conceptualization of the working life exposome (WLE), and initiate a process of deconstruction; and (d) explain how WLE-based epistemologies and methodologies can illuminate the *whole* of work and population well-being. The successful completion of this emerging discourse can significantly improve empirical research, policy, and action.

## Prevailing science can only go so far

The overall narrow focus of prevailing science has resulted in the underestimation of the totality, diversity, and complexity of multilayered and interdependent work and nonwork influences that impact people’s health and safety over their working lifespan and beyond. The aggregate impact of this underestimation has shaped traditional research in several ways:

First, theoretical foundations have been generally grounded in narrowly defined exposures unfolding primarily in the workplace, and within predominantly atheoretical, linear, individual-level, behavioral, and static conceptualizations. Linear causal thinking and single-level causal explanations, in particular, are preferred more often than not by contemporary work-health-safety conceptualizations. As for the former, conceptual frameworks are mainly informed by perspectives where the reciprocal and continual connections and movements among factors and their nested systems over time are absent. Regarding the latter, conceptual frameworks heavily rely on individual or intrapersonal factors at the expense of institutional, structural, organizational, and other meso-/macro-level factors necessary to more effectively understand persisting health challenges. These narrow approaches have naturally, in turn, led to the collection of data that regularly omit crucial information.

Second, these conceptualizations have, in turn, impaired methodological frameworks, leading to: (a) mainly cross-sectional, individual-level, and smaller-scale research designs; (b) data collection methods heavily based on traditional subjective approaches (eg, surveys) resulting in insufficient data; and (c) data that mainly include already known higher-risk exposures (eg, irregular shiftwork) unfolding in the workplace, and common or hard outcomes (eg, hospitalizations), with infrequent inclusion of potentially invaluable, rare, or lower-prevalence exposures and outcomes (2). Along these lines, methodological approaches have also been hampered by what is feasible in the context of the prevailing paradigm and practices, funding, time constraints, and available technology. And, in many cases, theoretical frameworks have also been adapted to these practical considerations.

While this is overall the case in international work-health-safety-well-being research, over the past couple of decades there have been several prospective studies originating mainly from northern Europe that have delved into connections between primarily the psychosocial work environment (eg, job strain) and cardiovascular and mental health outcomes (3). While these are invaluable, there is still an overall dearth of rigorous empirical research on associations between an array of crucial interconnected work (eg, chemical exposures, workhours, work environment) and health (eg, cancer) outcomes (3).

**Figure 1:**
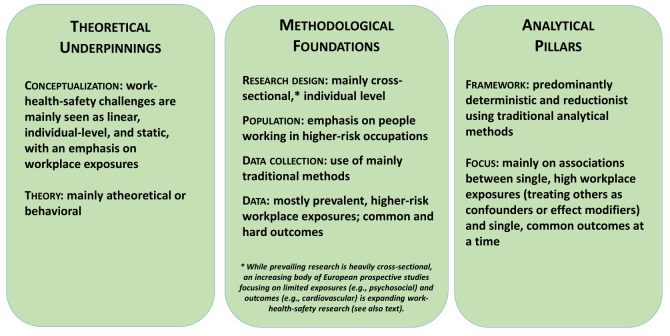
Tenets of traditional work-health-safety-wellbeing empirical research.

Third, based mainly on deterministic frameworks and a priori hypotheses, prevalent reductionist data analyses delve nearly exclusively into single, common workplace exposures – treating others as confounders or effect modifiers – by isolating individual or classes of related work-health-safety relationships at a time. These approaches only rarely examine mixtures of exposures, but even then, without fully disentangling their compositional complexity or by using rudimentary methods reflecting multiple exposures and/or components. Figure 1 outlines the tenets of traditional empirical research.

An example reflecting some of these limitations may be the evaluation of chemical hazards in the workplace related to risk for liver disease. "Material Safety Data Sheets" for chemicals report individual risks for specific chemical exposures, but there is virtually no information about additive or synergistic risk from exposures to multiple chemicals or exposures which damage the same end organ or system. A 2019 review assessing connections between vinyl chloride and liver diseases reported relationships documented in the literature, noting that very few studies considered other exposures related to liver diseases, and those were all treated as confounders in the relationship between vinyl chloride and liver disease (4). Vinyl chloride is an International Agency for Research on Cancer Group 1 carcinogen, with the relationship between vinyl chloride exposure and liver disease well-established, particularly in cases of long-term and high-level exposure (5). Vinyl chloride has been designated a carcinogen since 1978 and was officially classified as a Group 1 carcinogen in 1987, however nearly all research including research performed in the last 10 years, treats hepatitis, alcoholism, and exposure to other chemicals as confounders, instead of assessing the potential combined effects of these work and nonwork factors (6–8). One notable exception quantifies the synergistic relationship between vinyl chloride and alcohol use, which supports the idea that a more comprehensive and synergistic assessment may provide more insights (9). An accumulation of evidence across multiple studies is still required to robustly establish causation, however a more holistic research approach may expedite the trajectory from hypothesis to demonstrated causation.

Grounded in these assumptions, traditional research continues to have a limited understanding of how: (a) concurrent interactions of varied and multilayered work and nonwork factors can trigger synergistic biological processes throughout the working life and beyond; (b) critical lower-level or rare exposures can influence the health and safety of working people, given that common workplace exposures represent only the tip of the iceberg (2); and (c) complex biological pathways and mechanisms connect a multitude of diverse exposures with synergistic disease and/or injury burden.

Such limitations have perpetuated the incomplete understanding of mechanisms that trigger and/or exacerbate adverse biological and related outcomes, thus leading to flawed, monolithic policies that eventually reduce an action’s impact. Subsequently, such policies enable the continuation of conditions that are responsible for disease and/or injury burden across various cohorts of working people. Lastly, fast-evolving work organization (eg, expanding forms of precarious work) and increasing heterogeneity of working-life exposure patterns (eg, many different types of jobs throughout working life), along with ramifications of major epidemiological (eg, COVID-19 pandemic), geopolitical (eg, 2022 Russia-Ukraine war), and environmental (eg, climate crisis) events increasingly have adverse effects on working people. These have, thus in turn, started influencing empirical research. Because prevailing science can only go so far, epistemologies that can more fully address the totality, complexity, and spatiotemporality of such intractable health and safety challenges of working people would be invaluable.

## The *whole* of work and population health

Holistic epistemologies started getting traction in health research in the 1990s (10). This significant transition was based on earlier notions that epistemologies inclusive of social conditions (11) and the life course (12) are essential for delineating disease causation. Initially, most influential were those of ecosocial theories (13), multilevel dynamic perspectives (14), social production of disease (15), political economy of health (16), and social determinants of health (17), followed by those of the exposome (18) and syndemics (19). These epistemologies converged with scientific and technological breakthroughs – such as computing power, genome sequencing, big data, and multiomics – creating a path for the examination of health challenges as *wholes* broadly centered on complexity and exposome sciences. These epistemologies provide an opportunity to look at problems using multiple perspectives, studying micro (eg, behavioral patterns), meso (eg, environmental particulate matter), and macro issues (eg, unemployment), and understanding their interdependencies.

Originating outside the realm of health sciences, the explicit study of complex systems dates back to the 1970s. Complexity sciences highlight (a) the importance of interactions, self-organization, nonlinearity, emergence, and other properties, in understanding population health processes and outcomes, and (b) that the properties of complex systems cannot be understood from their components alone (20). Because complex systems require new analytical approaches, social network analysis (21) and simulation modeling (22) have provided the main antidote to reductionist research and are therefore more representative of overall population health reality.

Complex systems frameworks have not yet been comprehensively integrated into empirical research on the role of work in population health. Notable exceptions, albeit with limitations, are found in engineering (23), ergonomics (24), and social epidemiology (25), with systems theoretic accident model and processes (23), sociotechnical systems (24), and safe systems (26) approaches used primarily in workplace safety.

**Figure 2:**
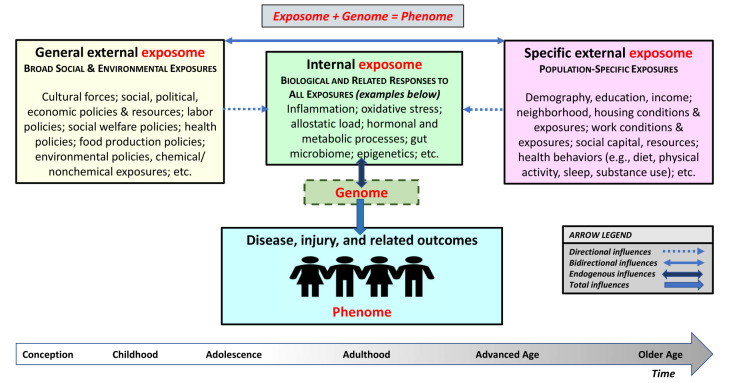
Simplified depiction of the role of the complex exposome in population health, safety, and well-being.

Exposome sciences, on the other hand, evolved from within health sciences, triggered by the recognition that the genome explains only a small proportion of the phenome (27). The exposome encompasses all nongenetic influences and biological responses throughout the lifetime, from conception onwards (28). Its domains are the: (a) general external exposome including sociocultural, political, economic, educational, labor, health, and environmental forces and exposures – applicable to all people; (b) specific external exposome including demographic, geographic, socioeconomic, chemical, physical, ergonomic, biological, occupational, and behavioral exposures – specific to a population and/or area; and (c) internal exposome including endogenous human processes resulting from foregoing exposures, being expressed along the lines of physiology and body morphology. The sum of these exposures and associated biological and related responses interacts with the genome and jointly generate the lifetime phenome (*exposome + genome = phenome*). Figure 2 depicts a high-level, heuristic illustration of the complex exposome.

Exposomic approaches, although still in their infancy, have gradually started being incorporated into population health research. This is not the case, however, with empirical work-health-safety research, with the only exception being the groundbreaking European Exposome Project for Health and Occupational Research (EPHOR) that delves into working life health (2, 29).

Not only can synergies of complexity and exposome epistemologies provide an avenue for integrating frequently fractured research along the lines of different diseases or injuries, but, most importantly, they can lead to unifying frameworks that can examine such challenges as *wholes* throughout the working life and beyond. Such synergies can be especially useful for delineating multiple concurrent exposures, where interdependencies abound, causality is uncertain (offering opportunities for discovery of unknown exposures), temporal sequence matters, social to biological interactions are critical, and subclinical markers are crucial for earlier diagnosis and timely action (30) – all rampant in work-health-safety milieux.

Learning from the accomplishments of these epistemologies, the combined use and/or development of holistic conceptualizations; longitudinal research designs; novel-technology-based utilization of better data; working-life exposomic databases (similar to *HapMap* project) (31); and novel mathematical, statistical, and computational methods hold promise to expand empirical research on work and population health, safety, and well-being. As such, these synergies can enhance current understanding of the totality and complexity of interacting, multifactorial work and nonwork exposures in disease and injury burden, thereby improving risk assessments, translation of science into practice, and prevention efficacy.

## The working life exposome

The modest successes of traditional research-based interventions along with ongoing changes in work organization as well as the foregoing innovations have collectively contributed to a slow but important epistemological transition in work-health-safety-well-being research toward more holistic approaches (32). EPHOR introduced the WLE as "all occupational and related nonoccupational exposures, with the latter including exposures that may be in/directly influenced by or interact with the working life in their relation to people’s health" (29). By expanding this definition, we view the WLE as the totality of an array of multilayered, interdependent work and relevant nonwork influences and exposures and associated biological responses and endogenous processes that concurrently impact people’s health, safety, and well-being from conception onwards, throughout and well beyond their working lifetime. The WLE presents an integrated function of internal/personal and external/structural influences and their biological and related consequences across people’s lives. Below we introduce the components, properties, and architecture of the WLE.

## Unpacking WLE components

In line with exposomic conventions, we provide an elaboration of the emerging WLE categorized as external and internal domains, with five closely interconnected, multilayered components.

The first two are work-related domains: "Exposures and factors" whilst working unfold within the traditional workplace as well as anywhere work takes place or activities we do in the broader context of work (eg, commuting to work, landscaping/agricultural work), and trigger immediate or delayed effects on the health, safety, and well-being of working people (33). Exposures fall broadly under the nature, content, design, load, conditions, and organization of work (eg, irregular schedules); organizational social work environment and associated psychological demands (eg, low job control); chemical and biological exposures (eg, toxic chemicals); remuneration (eg, wage types/amounts); sociodemographics (eg, race/ethnicity); and health-related behaviors (eg, smoking). "Work-defining exposures" originate outside work in the form of mainly labor-related, state and/or corporate policies that impact exposures whilst working, having a sustained bearing on the well-being of working people (34). These mainly include: state labor policies, such as minimum wage, unemployment benefits, etc; other state policies, such as occupational safety/health regulations and enforcement, workers’ compensation programs, etc; employment-based policies, benefits, and rights, such as guaranteed healthcare insurance, paid family and sick leave, etc; labor unions, including union access to workplaces, rights to form a union, unionization protections, collective bargaining, etc.; and market characteristics, such as occupational segregation (distribution of people across occupations based on demographic and/or racial/ethnic factors), labor market conditions, etc.

The next two are nonwork-related domains: "Broad nonwork exposures" occur outside the broad realm of work and are rooted in broader culture, social structures, and institutions. These multilayered domains directly or indirectly shape the life and well-being of all people, regardless of work status. They mainly involve sociocultural, political, economic, educational, labor, food, housing, health, and environmental forces, policies, and practices (eg, political processes, economic resources, urban planning, food production/access, chemical contaminants) that shape all exposures (35). "Nonwork exposures and factors of specific (eg, low-wage) working populations" also originate outside work and are particularly relevant for these cohorts of working people. These exposures result mainly from interacting exposures whilst working and work-defining exposures, are entrenched in broad nonwork exposures, and synergistically shape the life and overall well-being of these working populations (36). They may include the lack of vital social and economic resources (eg, education, social capital); residential neighborhood conditions (eg, poor housing, food deserts); physical and chemical exposures outside work; various health-related behaviors (eg, low-nutrition diet, sedentariness); and sociodemographic properties (eg, racial/ethnic discrimination, immigration).

**Figure 3:**
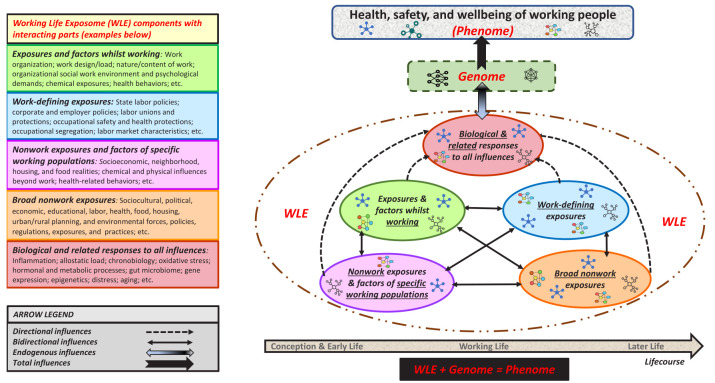
Heuristic portrayal of the role of working life exposome in the health, safety, and wellbeing of working people.

The final domain of "biological and related responses to all influences" are the cumulative embodiment of all foregoing domains and their interactions. They are endogenous processes, including chemicals from air, water, soil, and food and their metabolites, as well as endogenous chemicals produced by inflammation, chronobiology, oxidative stress, lipid peroxidation, infections, gut flora, epigenetics, and other natural processes. Figure 3 depicts a high-level heuristic portrayal of the role of WLE in the well-being of working people.

## Uncovering WLE architecture

WLE’s components are connected like networks, and each component includes several interacting, multilayered factors. The arrangement and connections of these varied networks (or network topology) can shape the WLE behavior over time, with consequences for the health and safety of working people. [In network nomenclature, components/factors are referred to as nodes and connections between the nodes as edges.] There are several important topological properties (37) that can define various substructures within the WLE. These include: (a) degree of a node or number of edges connecting to a node that can influence other characteristics (eg, node centrality) because degree distribution helps define whether a network is scale-free or not; (b) shortest path or shortest distance between any two nodes being used to model flow of information or transmission; (c) scale-free network where most nodes are connected to a low number of neighbors and there is a small number of high-degree nodes (hubs) providing high connectivity to the network; (d) transitivity or presence of tightly interconnected nodes that are more internally connected than they are with the rest of the network (topological clusters or communities); and (e) centrality or how important a node or an edge is for connectivity or the network’s information flow. These properties can affect other important processes, such as, preferential attachment of nodes or the more connected a node is, the more likely it is to receive additional links, which is quite useful in population health research.

Returning to the foregoing example of vinyl chloride and liver disease, let us imagine a spider web. Each junction where web strands come together is a node of a factor that may relate to our outcome; vinyl chloride exposure, hepatitis infection, alcohol use, but also factors like genetics, geographic location, access to health insurance, etc. These are all connected and perturbation of one of them ripples out through the entire web, interacting with all other nodes. Some advanced research has worked toward this end; for example, using time as a measure to understand and develop models describing multi-stage carcinogenesis (38). Historically, work-health-safety research has tried to isolate the interactions, but using the WLE approach, we want to measure the many potential synergies, beyond just looking at a single or even pairwise synergy that was assessed. In the next section we provide an illustrative example highlighting key network concepts and advantages.

Component/factor configuration can also lead to the emergence of complexity properties that can define the performance of the WLE over time. The WLE clearly meets three key conditions necessary for complexity to arise: (a) many interactions and feedbacks among many heterogeneous components/factors; (b) lack of central coordination; and (c) openness to the environment (39). These conditions have the potential to produce important collective patterns, such as those of nonlinearity, self-organization, adaptation, and emergence, among others, that describe complex systems (40). These patterns corroborate that the behavior of work-health-safety challenges cannot be easily inferred from their components/factors and properties alone. Especially because "more is different" (as entirely new properties emerge at each level of complexity) (41), this interaction of micro, meso, and macro patterns can produce emergent properties across different time scales. Time scale, which includes not only the period of one’s working life but also periconceptional and perinatal periods until the initiation of working life as well as post-working life, is particularly important for the WLE, highlighting the temporality of working life health. The empirical delineation of key WLE topological and complexity properties would be of great value for better understanding the intricacies and dynamics of persisting work-health-safety challenges.

## WLE at the center of working life health and safety

The emerging discussion of the WLE as a *whole* and its multifactorial components, properties, and architecture, further substantiates our thesis purporting that the health and safety of working people are attributable to a multitude of concurrent, complex, dynamic, and interacting influences, beyond the boundaries of the immediate workplace. And, while several work-defining and nonwork exposures and factors of specific working populations, along with common exposures whilst working, are often included in epidemiological research, they are rarely included in comprehensive population health and safety studies where all possible explanatory and/or mediating factors – along with relevant broad nonwork exposures – can be examined together. This omission has far-reaching ramifications, including the hindrance of the concurrent examination of the totality of potential exposures, their critical interactions, and resulting mechanisms and outcomes.

Ideally, a population-based birth cohort design would provide the basis for novel analysis to comprehensively determine causal relationships (42). For example, a large birth cohort of men and women from countries with different labor policies and levels of socioeconomic development, who eventually work in various occupations, are followed prospectively, while collecting all relevant exposures using diverse methods (including genetic information) as well as health and safety outcomes, would be the ideal study. Such a study would provide useful data for novel analyses to explore potential causality between exposures and outcomes.

This emerging analysis indicates that the WLE is marked by an interplay of simplicity and complexity that defines both its evolution and behavior over time. The foregoing preliminary mapping marks only the beginning of an ongoing and long empirical process that demands comprehensive data to fully understand, unravel, and ultimately validate the WLE.

Finally, these advancements in science, technology, and theory, along with commensurate training of researchers, will gradually enable more holistic studies by making the collection and analysis of more comprehensive data quite feasible. However, even these more holistic studies will oftentimes be restricted by similar budgetary and time constraints as many current studies do, which may oftentimes lead to decisions that are grounded in practical priorities.

## The WLE can elucidate the *whole* of work and population health

WLE epistemologies and associated technological, methodological, and analytical innovations can catalyze significant enhancements in empirical research by extenuating the intricacies of the essential *whole* of work and population well-being. Below, we outline potential contributions of WLE frameworks in all phases of empirical research.

On the theoretical front, WLE frameworks enable understanding of work–health–safety challenges as *wholes*, recognizing the totality, heterogeneity, and complexity of multilayered exposures that interact and concurrently influence the well-being of working people over their life course. Specifically, WLE frameworks: (a) provide the theoretical basis to identify interdependent work, work-defining, and a host of relevant nonwork exposures and factors that cumulatively shape the overall well-being of working people; (b) extenuate the importance of broader social conditions and their interactions with biological mechanisms, thereby providing insights into informative pathways linking exposures to well-being; (c) emphasize the temporal boundaries of exposures – that is, exposures unfolding from conception onwards, over the working lifespan and beyond; and (d) address ever-changing forms, conditions, and impacts of work over the life course.

These assumptions can also constitute key guiding propositions toward the development of a much-needed theory that accurately and comprehensively explains the health, safety, and well-being of working people. These conceptual/theoretical improvements will also define types of research questions asked, hypotheses stated, and data collected.

On the methodological front, WLE frameworks fully support the implementation of longitudinal research designs that consider the complexity, temporality, and unclear causality of health and safety challenges of working people over their life course. As such, WLE frameworks can contribute to: (a) the delineation of diverse occupational cohorts, with a particular interest in particularly vulnerable populations (eg, immigrant/minority workers, high-risk occupations), life stages (eg, older workers), and geographies (eg, economically depressed regions); (b) the employment of more potent data collection methods based on exposure biomarker technologies, geographical mapping and remote sensing technologies, smartphone applications and personal exposure sensors, and high-throughput molecular ‘omics’ techniques (43); (c) the collection of wide-ranging data that cover: (i) various multifactorial explanatory and/or mediating variables (eg, labor policies), including rare and/or lower-level exposures, and (ii) diverse biological and other related mechanisms, pathways, markers, and outcomes (eg, omics), without overlooking subtle or lower-prevalence outcomes (eg, how subclinical symptoms impact younger workers’ stress levels, thereby altering their immune status).

WLE-based frameworks can also enable the concurrent use of existing datasets (analogous to European exposome projects based on large-scale data pooled from multiple population, industry, and/or occupational cohorts) and various big data sources, including the exploitation of existing comprehensive and fragmented datasets, as well as the collection of key proxy measures (eg, family health history (44) to account for pre-working life exposures). Of course, the ongoing quest for comprehensive population-level data remains a significant challenge at the center of work-health-safety research.

Lastly, on the analytical front, WLE frameworks facilitate the application of both agnostic/untargeted (due to extensive knowledge gaps) and hypotheses-driven approaches. To start, inductive (agnostic) research, such as WLE-wide association studies, can contribute to identifying "causal signatures and fingerprints" of diverse exposures, thereby potentially uncovering new and/or confirming established mechanisms generating adverse outcomes. This can be followed by deductive research to test the plausibility of key hypotheses.

Because traditional statistical analysis is not designed to delineate compound, concurrent effects of many WLE components, the employment of a combination of novel analytical methods, taking advantage of stochastic analytical breakthroughs grounded in novel mathematical, statistical, and computational approaches (eg, machine learning, network analysis), can advance understanding of these complex challenges over the life course. Novel analytical methods employed in European (eg, Expanse) (45) and North American (eg, Hercules) (46) exposome projects can also be replicated and/or can catalyze the development of new analytical methods appropriate for evolving research needs.

**Figure 4:**
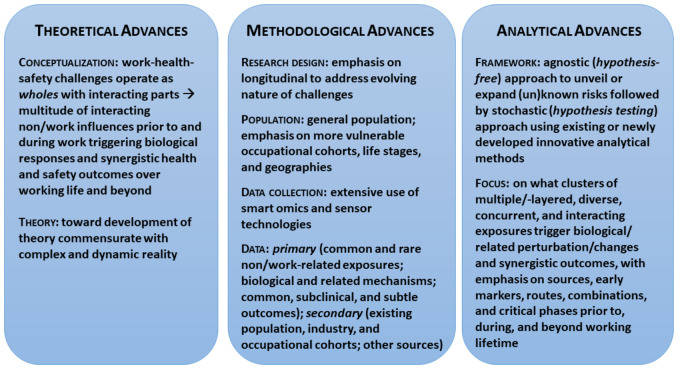
Tenets of emerging WLE-based work-health-safety-wellbeing empirical research.

This analytical process can facilitate the identification of key multilayered, diverse, and concurrent exposures that trigger biological perturbations and changes leading to synergistic adverse health and safety outcomes among working people, with an emphasis on their sources, early markers, routes, combinations, and critical phases prior to, during, and beyond the working lifetime. Figure 4 presents the tenets of emerging, WLE-based empirical research.

Case-in-point, "work precariousness" – an example of a foremost health and safety challenge (47) – can benefit from a WLE-grounded framework. We cannot fully understand "work precariousness" if we do not concurrently consider a host of multi-level and -layered factors, their interactions, and how they affect living and working conditions, which continually reshape the well-being of an increasing number of people who work under these conditions. Such intractable challenges are determined by, embedded in, and/or operate as complex systems consisting of a large number of disparate and multifactorial interacting components. Determining in advance a set of components/factors and properties that are studied separately (eg, unpredictable schedules and sleep quality), and then recombining this and other partial approaches of examining associations between various factors at a time, does not allow us to form an accurate picture of the *whole* – that is, deconstructing the complex mechanisms of the long-term negative effects of precarious work. Because of these characteristics, over the past decade there has been an increase of relevant empirical works grounded in prospective designs and the collection of richer, multilevel data (48–50).

This example corroborates that the presence of many interdependencies among diverse components/factors necessitates the concurrent examination of all relevant exposures unfolding not only during, but also prior to, precarious work, which aggregately have health ramifications beyond working time. This is necessary even if it means sometimes taking a *crude look at the whole* (coined in 1990 by renowned physicist Murray Gell-Mann), and then allowing possible simplifications to emerge from this approach. However, recent scientific advancements now allow a more complete examination of the *whole* of work-health-safety challenges which were not possible as recently as five years ago.

Especially because of the large number of multilayered factors involved in complex health and safety challenges, it is difficult to detect how their interactions organize collectively and how different effects may propagate within a large space of factors. For this reason, WLE-based epistemologies and methodologies that are designed to delve into the examination of concurrent interactions among multiple factors would be very useful. As such, network science approaches (51), for example, that inherently retain the full complexity of interactions while preserving the local environment of relevant factors, would be invaluable for examining concurrent interactions.

Along these lines, we use an actual but imperfect subsample of truck drivers (N=307), based on a larger trucker dataset (52), to illustrate how WLE-based network analysis can allow the study of clusters, paths, and other properties, without which these effects likely remain undetectable. [Dataset was compiled from multiple sources, including trucking company records, federal and state records, historical environmental data, and financial records, as well as primary trucker data (eg, demographics, work history, anthropometrics, health history, sleep health, life experiences).] Network analysis allows the exploration of a larger space of exposures and outcomes, where single connections will now be substituted by subsets of factors which are densely connected in the network representation. Because network structures (arrangement and connections of nodes) as defined by their topological properties (ie, clustering, centrality) can shape population health, network analysis can provide vital insights into the etiology of trucker health outcomes.

**Figure 5:**
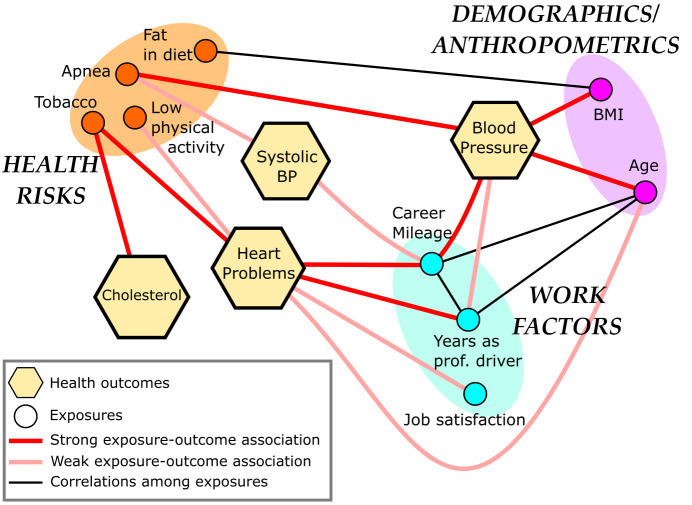
Trucker health network, created by a small sample of truckers.

Figure 5 provides a simplified proof-of-concept visualization of this network representation. Predictors (circles) include demographics/anthropometrics (eg, age, BMI), health risks (eg, sleep apnea, tobacco use, physical activity, fatty diet), and work factors (eg, miles travelled, years as trucker, job satisfaction). Trucker health outcomes (hexagons) include systolic blood pressure, cholesterol, diagnosed heart problems, and hypertension. For each health outcome, we selected all truckers in the subsample whose outcome value exceeds established high levels (eg, cholesterol > 240). We then compared the distribution of each possible predictor value in this subsample with the distribution of the same predictor in the general population through standard Kolmogorov-Smirnov techniques. A statistically significant difference between these distributions indicated that the predictor can be associated with the outcome, while the Kolmogorov-Smirnov value provided the strength and direction of this association. We also calculated linear correlations between predictors through the Pearson coefficient between all predictor pairs.

In this network visualization, we only represent the strongest connections, and use red lines to indicate a positive association (larger values increase the probability for a given outcome). We use thicker/thinner line weights to indicate a stronger or weaker extent of association, as found by comparing the distance between the distribution of a factor in the dataset with distribution of the same factor in a null trucker dataset not involved in trucker health outcomes. Gray lines connect exposures/factors, so that the existence of a line indicates statistically significant linear correlation between any two nodes.

Even with a small sample size and a few variables, we get meaningful information out of this trucker health network. For example, demographic/anthropometric factors, such as age and BMI, contribute significantly to hypertension, health risks are associated with all four trucker health outcomes, and work factors are highly correlated with diagnosed heart problems and hypertension. We also recover some trivial correlations, such as between a fatty diet and BMI and age with career mileage. These results provide clear clues as to how we can explore interdependencies grounded in WLE-based analytical frameworks. For example, clustering analysis of a large-scale network can identify specific factors or groups of factors that all act together to influence a health outcome, even if each factor is not significant on its own. Similarly, centrality measures can reveal a group of variables, whose location in the network may affect multiple health outcomes or connect otherwise disjointed network areas. Results of this nature and their implications are not possible without a holistic network consideration.

We expect that network analytical strengths will increase multifold as we examine combinations of WLE factors that have not been studied together thus far. For example, co-occurring work and nonwork exposures – nodes representing federal (eg, hours-of-service) or trucking (eg, by-the-mile pay) policies, company-paid benefits (eg, health insurance), truckers’ working life (eg, work-life balance), trucker type (eg, company driver/owner operator), environmental policies (eg, truck stop air quality), or geospatial factors (eg, parking availability along frequent routes) – will reveal topological key properties to help in more fully understanding, explaining, modeling, and predicting the emergence and dynamics of truckers’ health risks.

Lastly, given these novel but very pragmatic directions for work-health-safety-well-being research, the emerging WLE framework, especially in this early phase, is faced with various challenges. Key among these are issues regarding the: (a) assessment of multiple, multidimensional, heterogeneous, and oftentimes hard to access mixtures of exposures over the life course as well as based on digital apps, data sources, (spatial) models, use of AI in health, and other innovations; (b) collection of particularly large and complex datasets (including contrasts in exposures) as well as their longitudinal analyses; and (c) assessment of causality because of confounding, reverse causation, and other uncertainties (30). To tackle such challenges, which are typical of observational and exposomic designs and which are also present in WLE–based epidemiological research, there have been evolving and synergistic scientific and technological breakthroughs. On the assessment front, a combination of proxy exposures, different methods of data collection and tools, along with novel technologies (eg, sensors, GIS, high-throughput ‘omics’) can help identify exposure biomarkers and even allow integration of varied exposures to single measures (30). On the analytical front, because of dealing with high dimensionality, studying the combined effects of exposures and their interactions, and integrating causal pathways as well as high-throughput omics layers, more novel analytical methods such as mediation analysis, g-computation methods, and causal random forest can make significant contributions to this end (53, 54). Finally, on the causality front, among others, "triangulation" approaches (using diverse computational and statistical advances to address one question) and involvement of novel "omic" technologies, combined with broad data sharing and cross-study collaborations offer substantive opportunities to strengthen causal inference (54, 55).

## Future directions

Despite ongoing analytical, technological, and theoretical advances, prevailing empirical research continues to practically underestimate the complex, systemic, and dynamic characteristics of work-health-safety-well-being challenges. Herein we elaborate the overdue need for the systematic advancement and proliferation of a new holistic paradigm to disease etiology that considers the simultaneous exposure to mixtures of multiple factors, grounded in the comprehensive WLE and associated theories, epistemologies, methodologies, and analytical approaches.

The defining role of work in human health, enduring impasses of traditional empirical research, overall ineffective population-level interventions, and a confluence of scientific and technological advances point toward the need for more impactful ways of examining the health and safety challenges of working people over their life course. Albert Einstein said, "we cannot solve our problems with the same thinking we used when we created them," while Thomas S. Elliot contended "only those who will risk going too far can possibly find out how far one can go". Although in an early stage, the emerging WLE presents itself as a novel and potent epistemological, methodological, and analytical framework that can enable the gradual unpacking of the indispensable *whole* of work and population health, safety, and well-being. It provides an invaluable roadmap to guide the challenging empirical endeavor of the gradual mapping, operationalization, and measurement of important properties of both occupation-specific and population WLE. This emerging paradigm shift has the potential to move empirical research on work and population health, safety, and well-being to the frontier of science, and eventually enable more impactful policies and actions.
